# Cervical Dysplasia and High-Risk Human Papillomavirus Infections among HIV-Infected and HIV-Uninfected Adolescent Females in South Africa

**DOI:** 10.1155/2014/498048

**Published:** 2014-10-20

**Authors:** David H. Adler, Melissa Wallace, Thola Bennie, Megan Mrubata, Beau Abar, Tracy L. Meiring, Anna-Lise Williamson, Linda-Gail Bekker

**Affiliations:** ^1^Department of Emergency Medicine, University of Rochester, 601 Elmwood Avenue, P.O. Box 655, Rochester, NY 14642, USA; ^2^Desmond Tutu HIV Centre, Institute of Infectious Diseases & Molecular Medicine, Faculty of Health Sciences, University of Cape Town, Anzio Road, Observatory, Cape Town, South Africa; ^3^Institute of Infectious Diseases & Molecular Medicine and Division of Medical Virology, Faculty of Health Sciences, University of Cape Town, Anzio Road, Observatory, Cape Town, South Africa; ^4^National Health Laboratory Service, Groote Schuur Hospital, Cape Town, South Africa

## Abstract

*Background.* HIV-infected adolescents may be at higher risk for high-grade cervical lesions than HIV-uninfected adolescents. The purpose of this study was to compare the prevalence of high-risk HPV (HR-HPV) infections and Pap smear abnormalities between these two groups. *Methods.* In this cross-sectional study, we compared the HPV DNA and Pap smear results between 35 HIV-infected and 50 HIV-uninfected adolescents in order to determine the prevalence of HR-HPV genotypes and cervical cytological abnormalities. Comparisons were made using Pearson *χ* ^2^ and independent-samples *t*-tests analyses, and associations between demographic and behavioral characteristics and HPV infections were examined. *Results.* HIV-infected participants were more likely to be infected with any HPV (88.6% versus 48.0%; *P* < 0.001) and with at least one HR-HPV (60.0% versus 24.0%; *P* = 0.001), and to have multiple concurrent HPV infections (68.6% versus 22.0%; *P* < 0.001). HPV 16 and 18 were relatively underrepresented among HR-HPV infections. Abnormal Pap test results were more common among HIV-infected participants (28.8% versus 12.0%; *P* = 0.054). A history of smoking was associated with HR-HPV infection. *Conclusions.* HIV-infected adolescents have an increased risk of infection with HR-HPV and of Pap test abnormalities. The majority of HR-HPV infections among our participants would not be prevented by the currently available vaccinations against HPV.

## 1. Introduction

Human papillomaviruses (HPV) cause cervical cancer [1]. The global burden of cervical cancer falls disproportionately on the developing world where 86% of cases occur [2]. Cervical cancer is an AIDS-defining illness [3] and the global distributions of HIV and cervical cancer covary closely, with the highest rates found in sub-Saharan Africa [2, 4]. In South Africa, cervical cancer is the most common cause of cancer-related death among women [5].

Women who are infected with HIV are at increased risk of dying from cervical cancer [6]. They are more likely to have persistent infections with high-risk HPV genotypes (HR-HPV) and to have progressive precancerous lesions [7, 8]. Due to this increased risk, cervical cancer screening guidelines for HIV-infected and HIV-uninfected women differ [9, 10].

Screening guidelines also differ between adults and adolescents. Due to the high rate of spontaneous clearance of low-grade cervical lesions and the risks associated with over-treatment among adolescents, it is recommended that screening does not begin until the age of 21 [11, 12]. HIV-infected adolescents, however, constitute a special high-risk population for whom questions remain regarding the natural history of HPV infection and low-grade cervical lesions and the difference in risk for high-grade cervical lesions between them and their HIV-uninfected counterparts. The optimal approach to cervical cancer screening for HIV-infected adolescents, including the potential role for HPV DNA testing, is uncertain. Research conducted with an HIV-infected adolescent population is necessary to address these questions and allows for inquiry into HPV infections and their sequelae during the early years of sexual activity.

We compared the HPV DNA and Pap smear results between 50 HIV-uninfected and 35 HIV-infected adolescent females from Masiphumelele, South Africa, in order to determine the prevalence of HR-HPV, multiple concurrent HPV infections, and cervical cytological abnormalities. We examined associations between these results and potential risk factors including sexual activity, contraception use, and smoking.

## 2. Materials and Methods

### 2.1. Study Participants

Between October 2012 and February 2014 we conducted a cross-sectional prevalence study in which cervical specimens for Pap smear and self-collected vaginal swabs for HPV DNA testing were collected from 50 HIV-uninfected and 35 HIV-infected sexually active South African adolescent females aged 17–21 (*M* = 19.06 years; S.D. = 1.48; IQR = 18.00–20.00). While definitions of the age range that constitutes adolescence vary, we defined it in accordance with the American Academy of Pediatrics that categorizes late adolescence to extend to the age of 21 [13]. Exclusion criteria included history of vaccination against HPV and history of cervical surgery. In order to confirm HIV status all study participants underwent HIV testing on the same date as Pap smear and HPV DNA testing. Study participants were recruited from a youth community center in Masiphumelele, a township in Cape Town, South Africa.  Table 1 describes the demographic and behavioral characteristics of our cohort by HIV status.

All participants signed informed consent (aged 18 and older) or signed adolescent assent documents (aged 17) to accompany parental consent forms in order to participate in the study. This study was approved by the Research Subjects Review Board at the University of Rochester and the Human Research Ethics Committee at the University of Cape Town.

### 2.2. Laboratory

For self-sampling, patients were instructed to insert a Dacron swab high into the vagina and twirl it for 10 seconds. Self-sampling was conducted in private. Samples were stored in Digene transport medium and DNA was extracted using the MagNA Pure Compact Nucleic Acid Isolation Kit (Roche Diagnostics). HPV genotyping was conducted using Roche's Linear Array HPV Test. This kit detects 37 HPV genotypes [14] including all oncogenic HPV types identified by the International Agency for Research on Cancer (IARC) [15]. We defined HR-HPV to include 13 genotypes designated by IARC to have sufficient evidence to cause cervical cancer (types 16, 18, 31, 33, 35, 39, 45, 51, 52, 56, 58, and 59) and to have strong mechanistic evidence for cervical cancer (type 68) [15].

Pap smear results were reported in accordance with the Bethesda system [16]. All positive Pap smears that were identified by cytotechnologists were sent for review by a pathologist. Women with incidentally identified infections on Pap smear (e.g.,* Trichomonas*) were referred for appropriate treatment. Most recent CD4 test results were obtained from the local government clinic where HIV-infected participants receive care.

### 2.3. Statistics

HIV-infected and HIV-uninfected youth were compared using Pearson *χ* ^2^ and independent-samples *t*-tests analyses, and associations between demographic/behavioral characteristics and HPV infections were examined using Spearman correlations. All analyses were performed using IBM SPSS 22.0.

## 3. Results

We found a large burden of HPV infection in our study cohort. Overall 64.7% of participants had at least one HPV infection and 38.8% had at least one HR-HPV infection. Multiple concurrent HPV infections were common with 41.2% of all participants testing positive for two or more HPV genotypes (63.6% of HPV-infected participants were infected with greater than one genotype). Multiple concurrent HR-HPV infections were found in 24.7% of all participants and 63.6% of HR-HPV-positive participants were infected with two or more HR-HPV genotypes. Eleven out of the 85 participants (12.9%) had five or more concurrent HPV infections detected. Likewise, 24.2% of HR-HPV positive participants had three or more concurrent HR-HPV infections.

Non-vaccine HR-HPV genotypes (those other than 16 and 18) were notably common among our cohort. Among HR-HPV positive participants, only 12.1% were infected with exclusively vaccine genotypes, while 51.5% were infected with exclusively non-vaccine genotypes (36.4% were infected with a combination of vaccine and non-vaccine genotypes).

Numerous significant differences in HPV infection were identified between the HIV-infected and HIV-uninfected groups. HIV-infected participants were more likely to be infected with any HPV, to be infected with at least one HR-HPV, and to have multiple concurrent HPV infections. Although HPV 16 was more prevalent in the HIV-infected group (20.0% versus 6.0%; *P* = 0.049), among all participants with one or more HR-HPV infections, HIV-infected individuals were more likely to be infected with a non-vaccine HR-HPV genotype (*P* = 0.006).  Table 2 summarizes the epidemiology of HPV in our study cohort.

Overall, 18.8% of our cohort had abnormal Pap tests. Pap smear abnormalities were found to be more common among HIV-infected participants than their HIV-uninfected counterparts. No study participants were found to have high-grade squamous intraepithelial lesions (HSIL). There were two HIV-infected youths observed with atypical squamous cells—cannot rule out HSIL (ASC-H) and six with low-grade squamous intraepithelial lesions (LSIL) compared to zero and two, respectively, among HIV-uninfected youth.  Table 3 summarizes Pap smear results for our cohort. Infection with one or more HR-HPV genotypes and the presence of multiple concurrent HPV infections were positively associated with cytological abnormality on Pap smear (*P* = 0.006 and *P* = 0.002, resp.).

Among the HIV-infected participants, 22.9% were receiving antiretroviral medications (ARVs) at the time of testing. The average CD4 count among all HIV-infected participants was 490/mm^3^ (IQR = 322–539; CD4 counts were not available for 8 participants). The average CD4 for those on ARVs was 301 and for those not on ARVs was 557 (*P* = 0.012). We did not identify any significant relationship between ARVs and HPV infection. Likewise, CD4 count was not found to be significantly associated with HR-HPV infection or abnormal Pap test.

Among our bivariate analyses, we found the older members of our cohort to have a greater number of multiple concurrent HR-HPV infections (*P* = 0.049). A history of smoking was positively associated with HR-HPV infection (*P* = 0.024). Methods of contraception used, number of lifetime sexual partners, and number of sexual partners reported in the six months prior to enrollment were not significantly associated with HPV infection.

## 4. Discussion

Our results show an increased risk of infection with HR-HPV and associated Pap smear abnormalities among HIV-infected adolescents compared to their HIV-uninfected counterparts. This is consistent with previous data from South Africa. A recent report comparing the distribution of HPV genotypes between HIV-infected and HIV-uninfected South African women aged 17–65 years found differences in HR-HPV prevalence across all age groups including 17–19-year-olds who were found to have an HR-HPV prevalence of 75% and 60%, respectively [17] (compared to our 60% and 24%; *P* = 0.001). Marais et al. have reported similar differences in HR-HPV prevalence between HIV-infected and HIV-uninfected South African women from an older cohort (mean age: 35) [18]. The longitudinal data being obtained from our young cohort will be essential to assess differences in the risk of HR-HPV persistence, as well as the incidence, persistence, and progression of cervical dysplasia in this age group.

Although identifying the HR-HPV genotypes associated with cases of invasive cervical cancer provides the most critical information regarding the proportion of cases preventable by vaccination, the high relative prevalence of non-vaccine HR-HPV compared to the vaccine genotypes in our cohort does demonstrate that the currently available vaccines against HPV would not prevent the majority of HR-HPV infections among our study population. Still, without longitudinal data demonstrating incident and/or persistent cervical disease associated with non-vaccine HR-HPV infection, the significance of this observation is uncertain.

HIV-infected women in sub-Saharan Africa are among the groups at highest risk of developing cervical cancer. Although adolescents are generally at low risk for cervical cancer [19], those who are infected with HIV may not be. Several investigators have observed a shorter preinvasive stage and an earlier age of diagnosis of invasive cervical cancer among this group [20, 21]. Low-grade cervical lesions are more likely to persist and progress among HIV-infected adolescents [22]. Moscicki et al. found the incidence of HSIL among HIV-infected adolescents to be 21.5% over a follow-up period of approximately 3.5 years [23]. While cervical cancer prevention efforts may appropriately focus on vaccination, the need for effective secondary prevention efforts will not abate. In many high-risk areas, including South Africa, access to vaccination is notably limited. Moreover, a large proportion of HIV-infected women have already been exposed to one or more HR-HPV and are expected to benefit less from vaccination. Finally, current screening guidelines do not change based on vaccination status [11, 12].

All cervical cancer screening strategies endeavor to identify cervical dysplasia prior to it reaching an invasive stage while also protecting women from the risks of overtesting and overtreatment. Tremendous efforts have been made to identify cost-effective approaches to the follow-up of negative Pap tests and low-grade Pap smear abnormalities, resulting in recommendations that utilize HPV DNA testing to extend intervals between Pap tests [11, 12]. These recommendations do not extend to HIV-infected women or adolescents for whom annual Pap tests are still recommended. The American College of Obstetricians and Gynecologists and The US Public Health Service's guidelines for prevention of cervical cancer in HIV-infected women recommend two Pap smears in the first year after diagnosis of HIV infection and annually thereafter regardless of age [9, 10]. HPV DNA testing, although recently recommended for HIV-uninfected women aged 30 and older [11, 12], has not been integrated into screening guidelines for HIV-infected women of any age. Still, investigators have demonstrated the potential utility of HPV DNA testing for HIV-infected women [24, 25]. Longitudinal studies with our cohort or similar cohorts may show that HPV DNA tests, when negative for HR-HPV, safely allow for extension of this testing interval.

In this study, self-collected vaginal swabs were used to provide samples for HPV DNA testing. Agreement between the results from clinician-collected and self-collected specimens has been reported to be high in several studies [26], even among adolescents [27]. In addition, self-sampling may offer greater acceptability and has a potential role for screening in low-resource settings or among remote populations where clinic-based screening is not feasible.

Our study setting has very high density of HIV and HPV infection, providing a unique opportunity to investigate cervical cancer risk among HIV-infected adolescents. Still, our study has several limitations. Although numerous significant data were found, our sample size is relatively small and may be insufficient to identify real differences between study groups. Our current sample size limits the types of multivariate analyses that could be performed to accompany the descriptive statistics and bivariate correlations presented. Subsequent research should seek to examine differences across HIV status while accounting for demographic and behavioral variables. Only 22.9% of our HIV-infected participants were on ARVs and our cohort had relatively high CD4 counts so we are unable to correlate our findings with levels of immunosuppression. Previous research has shown that more advanced HIV disease is strongly associated with increased cumulative HPV prevalence, more advanced cervical dysplasia, and an increased rate of progression of cervical disease [7, 8, 28–30]. The differences found between our HIV-infected and HIV-uninfected groups may be in part due to immunologic or behavioral factors that were not assessed in this study. Finally, our study cohort was drawn from a small geographical area, possibly limiting the generalizability of our findings.

## 5. Conclusions

Our results underscore the overall high-risk for HPV-related cervical disease in our study population and the significant potential difference in risk for cervical cancer between HIV-infected and HIV-uninfected young women in South Africa. The majority of HR-HPV infections among our study participants would not be prevented by the currently available vaccinations against HPV although the impact of these infections on incident advanced cervical disease is not clear. An optimal, evidence-based approach to cervical cancer screening for HIV-infected adolescents may include the use of HPV testing which has the potential to allow for an increase in the interval between Pap tests. This would have particular benefits in low-resource settings such as Masiphumelele. Longitudinal studies with this and similar cohorts are required to quantify the difference in risk for high-grade cervical lesions between HIV-infected and HIV-uninfected adolescents and to determine if HPV testing can be effectively integrated into a cervical cancer-screening program for adolescents with HIV.

## Figures and Tables

**Table 1:** Demographic and behavioral variables by HIV status^a^.

	HIV-infected *n* = 35		HIV-uninfected *n* = 50		*P* value
	Frequency (%)	M (S.D.)	Frequency (%)	M (S.D.)	
Age (years)		19.94 (1.11)		18.44 (1.40)	<0.001
Ever used tobacco	5 (14.7)		3 (6.0)		0.169
Number of sexual partners—lifetime	1 = 4 (11.4) 2–5 = 27 (77.1) >5 = 4 (11.4)		1 = 3 (6.0) 2–5 = 44 (88.0) >5 = 3 (6.0)		0.414
Number of sexual partners—past 6 months	1 = 33 (94.3) 2–5 = 2 (5.7)		1 = 47 (94.0) 2–5 = 3 (6.0)		0.956
Frequency of sex (days per month)		7.50 (5.42)		5.68 (4.41)	0.095
Current contraception^b^					
None	1 (2.9)		1 (2.0)		0.781
Condom	33 (97.1)		41 (82.0)		0.036
Injection	17 (50.0)		32 (64.0)		0.201
Pill	1 (2.9)		4 (8.0)		0.336

^a^Percentages in table are based on nonmissing data.

^b^Percentages exceed 100% because many participants use more than one contraceptive method.

**Table 2:** Epidemiology of HPV by HIV status.

	HIV-infected *n* = 35 Frequency (%)	HIV-uninfected *n* = 50 Frequency (%)	*P* value
Any HPV	31 (88.6)	24 (48.0)	<0.001
Any LR-HPV	29 (82.9)	17 (34.0)	<0.001
Any HR-HPV	21 (60.0)	12 (24.0)	0.001
Vaccine HR-HPV			
HPV 16	7 (20.0)	3 (6.0)	0.049
HPV 18	5 (14.3)	2 (4.0)	0.090
HPV 16 and/or 18	11 (31.4)	5 (10.0)	0.013
HPV 16 and/or 18 only (non-vaccine HR-HPV not present)	3 (8.6)	1 (2.0)	0.375
Non-vaccine HR-HPV			
HPV 31	1 (2.9)	0 (0.0)	0.229
HPV 33	1 (2.9)	0 (0.0)	0.229
HPV 35	5 (14.3)	1 (2.0)	0.030
HPV 39	2 (5.7)	2 (4.0)	0.713
HPV 45	9 (25.7)	0 (0.0)	<0.001
HPV 51	3 (8.6)	3 (6.0)	0.649
HPV 52	4 (11.4)	2 (4.0)	0.188
HPV 56	2 (5.7)	0 (0.0)	0.087
HPV 58	1 (2.9)	1 (2.0)	0.798
HPV 59	1 (2.9)	1 (2.0)	0.798
HPV 68	7 (20.0)	3 (6.0)	0.049
Any non-vaccine HR-HPV	18 (51.4)	11 (22.0)	0.005
Non-vaccine HR-HPV only (HPV 16 and 18 not present)	10 (28.6)	7 (14.0)	0.098
Multiple concurrent HPV	24 (68.6)	11 (22.0)	<0.001
Multiple concurrent HR-HPV	15 (42.9)	6 (12.0)	0.001

**Table 3:** Pap test results by HIV status.

	HIV-infected *n* = 35 Frequency (%)	HIV-uninfected *n* = 50 Frequency (%)	*P* value
Abnormal Pap test	10 (28.6)	6 (12.0)	0.054
Pap test results			0.058
ASC-H	2 (5.7)	0 (0.0)
ASCUS	2 (5.7)	4 (8.0)
LSIL	6 (17.1)	2 (4.0)
Normal	25 (71.4)	44 (88.0)

ASC-H: atypical squamous cells—cannot rule out high-grade intraepithelial lesion; ASCUS: atypical squamous cells of undetermined significance; LSIL: low-grade squamous intraepithelial lesion.
